# Highly sensitive detection of CYFRA21-1 with a SERS sensing platform based on the MBs enrichment strategy and antibody-DNA-mediated CHA amplification

**DOI:** 10.3389/fbioe.2023.1251595

**Published:** 2023-08-10

**Authors:** Xiaotao Bao, Shiyi Wang, Xiaoyan Liu, Guang Li

**Affiliations:** ^1^ Department of Otorhinolaryngology Head and Neck Surgery, The Affiliated Hospital of Yangzhou University, Yangzhou University, Yangzhou, China; ^2^ Department of Otorhinolaryngology Head and Neck Surgery, Xishan People’s Hospital of Wuxi City, Wuxi, China

**Keywords:** surface-enhanced Raman scattering, catalytic hairpin self-assembly, biomarker, laryngeal carcinoma, diagnosis

## Abstract

Laryngeal carcinoma (LC) is the second most common malignant tumor of the head and neck. Due to its insidious nature, most patients have developed to the middle and late stages by the time they are diagnosed, missing the best treatment period. Thus, early detection, diagnosis and treatment are crucial to improve the prognosis of LC and enhance the quality of life of patients. In this study, a surface-enhanced Raman (SERS) sensing platform was developed by combining the magnetic beads (MBs) enrichment strategy and the antibody-DNA-mediated catalytic hairpin self-assembly (CHA) signal amplification technology. 4-Mercaptobenzoic acid (4-MBA) and hairpin DNA 1 (hpDNA1) were modified onto the surface of gold nanobipyramids (GNBPs) as SERS nanotags. Hairpin DNA 2 (hpDNA2) modified MBs were used as capture nanoprobes. Under the action of CHA and magnet-induced MBs enrichment, GNBPs can be assembled on the surface of MBs, forming high-density “hot spots” for the SERS signal enhancement. The results showed that the SERS sensing platform has the advantages of high sensitivity, high specificity and high reproducibility, with the limit of detection (LOD) low to pg/mL level. The expression level of CYFRA21-1 in serum of LC patients and healthy controls was successfully detected by the SERS sensing platform. The accuracy of the SERS results was verified by enzyme linked immunosorbent assay (ELISA). Therefore, this SERS sensor can be used for the detection of CYFRA21-1 in serum, providing a simple and reliable new method for the early diagnosis of LC.

## 1 Introduction

In recent years, the incidence of malignant tumors has shown a rising trend, which seriously threatens the life and health of human beings. According to relevant data, the mortality rate of malignant tumors has increased by 83.1% compared with the mid-1970s, and the incidence rate of tumors has increased at a rate of 3%–5% per year (Asahi et al., 2018; Li et al., 2020; Ohashi et al., 2022; Hosny et al., 2023). Laryngeal carcinoma (LC) is the second most common malignant tumor of the head and neck, and diseases such as atypical hyperplasia of the larynx, leukoplakia of the vocal cords, and papilloma are prone to transform into laryngeal cancer (Wiegand et al., 2020; Li and Cheng, 2021; Sahin et al., 2022; Teng et al., 2022). The 5-year relative survival rate for patients with LC varies widely according to tumor site and stage. However, it should be noted that the 5-year survival rate of LC has been proved to decline gradually over the past 40  years, from 66% to 63% since most patients have developed to the middle and late stages by the time they are diagnosed, missing the best treatment period (Ono et al., 2020; Miskiewicz-Orczyk et al., 2021; Mur et al., 2022; Zhou et al., 2022). Therefore, early detection, diagnosis and treatment are crucial to improve the prognosis of LC. Although microlaryngoscopy combined with pathological tissue biopsy is still the “gold standard” for the diagnosis of LC and precancerous laryngeal lesions, this method requires special equipment and specialized technicians, and the examination is expensive and economically costly, which limits its further use in the population. In addition, due to the special location of LC and the lack of specific symptoms such as hoarseness, foreign body sensation in the throat, cough and dyspnea in the early stage, laryngoscopic biopsy may easily damage the surrounding vocal cords and other tissues. Therefore, establishment of simple, non-invasive and highly sensitive detection methods for early diagnosis of LC has become an urgent problem in the current research field.

Biomarker is a category of substances produced due to the tumor abnormalities or tumor stimulation of the host and are important references for assessing tumorigenesis, progression, and prognosis (Matzke and Watson, 2020; Polley and Dignam, 2021; Pilvenyte et al., 2023). Cytokeratin 19 fragments (CYFRA21-1) is a soluble fragment of cytokeratin 19 (Zhu et al., 2022). During epithelial tissue or cell lysis or carcinoma necrosis shedding, a large amount of CYFRA21-1 is released into the circulation in the form of lysed fragments, resulting in abnormally high CYFRA21-1 level. Doweck et al. (1995) measured the expression level of CYFRA21-1 in 250 serum samples by IRMA method and showed that 60% of patients with head and neck squamous carcinoma had increased serum CYFRA21-1 level, and the serum CYFRA21-1 level was positively correlated with tumor stage and lymph node metastasis. Kuropkat et al. (2002) monitored follow-up patients and found that CYFRA21-1 level was consistently elevated before the start of tumor treatment, decreased after tumor resection, and increased again when the tumor recurred, suggesting that CYFRA21-1 is involved in the development and evolution of LC. Currently, the analytical process of traditional methods such as enzyme linked immunosorbent assay (ELISA) and protein immunoblotting assay involves a lot of complex manual operations and presents poor reproducibility and low sensitivity (Reed and Park, 2010; Ahirwar et al., 2022). Therefore, it is quite necessary to develop a new assay method with simple operation, high sensitivity and specificity.

Surface-enhanced Raman scattering (SERS) is a detection technique that uses light excitation of electrons on the surface of rough metal nanostructures to produce localized surface plasmon resonance (LSPR) to achieve significant amplification of Raman signals (Wu et al., 2017; Kim et al., 2021; Hu et al., 2022). SERS has the advantages of excellent sensitivity, noninvasive detection capability and unique fingerprint effect (Chen et al., 2010; Hou et al., 2015; Yu et al., 2021). In addition, SERS is not interfered by the signal of aqueous solution and can detect samples in liquid state, which is ideal for the detection of clinical biological samples. Gold nanobipyramids (GNBPs) are anisotropic nanostructures with excellent optical properties. GNBPs have high monodispersity and narrow peak linewidth, making them extremely sensitive to dielectric environments (Yang et al., 2021). The LSPR wavelength of GNBPs can be adjusted by changing the aspect ratio, even to the near-infrared region The sharp tips of GNBPs exhibit significant local electric field enhancement, which can effectively enrich plasma “hot spots”. In addition, aggregated GNBPs can generate more abundant “hot spots” in adjacent nanogaps, promising single-molecule detection.

Nowadays, protein-based biomarker SERS assays rely mainly on an antibody sandwich strategy. Although the operation is simple and has reliable specificity; however, due to the extremely low concentration of biomarkers in the sera of early LC patients, only rely on the antibody sandwich strategy cannot meet the demand for highly sensitive detection. In recent years, the emergence of the aptamer strategy has broken the traditional understanding of nucleic acids which can only act as carriers of genetic information storage and transmission. The strategy of using aptamer as a recognition molecule combined with nucleic acid signal amplification has met the demand for highly sensitive detection of target proteins. However, the current aptamer library is relatively scarce, many proteins still lack satisfactory aptamers, and the aptamer strategy is severely constrained by its sequence diversity. Therefore, the development of a simple and universal detection strategy has become a breakthrough to expand the clinical application of SERS. Catalytic hairpin assembly (CHA) is a nonenzymatic amplification technique which can provide a useful mean in amplifying and transducing signals from nucleic acid analytes in recent years. For typical hairpin assembly amplification, the two hairpin DNA sequence 1 (hpDNA1) and hairpin DNA sequence 2 (hpDNA2) are in stably coexist in solution. When the target added, the hairpin structure of hpDNA1 is opened, subsequently hpDNA2 hybridizes with the exposed complementary sequence of hpDNA1 to displace the target and hpDNA1-hpDNA2 complexes are formed. Then, the displaced target triggers another circulation DNA assembly reaction to achieve signal amplification.

**FIGURE 1:**
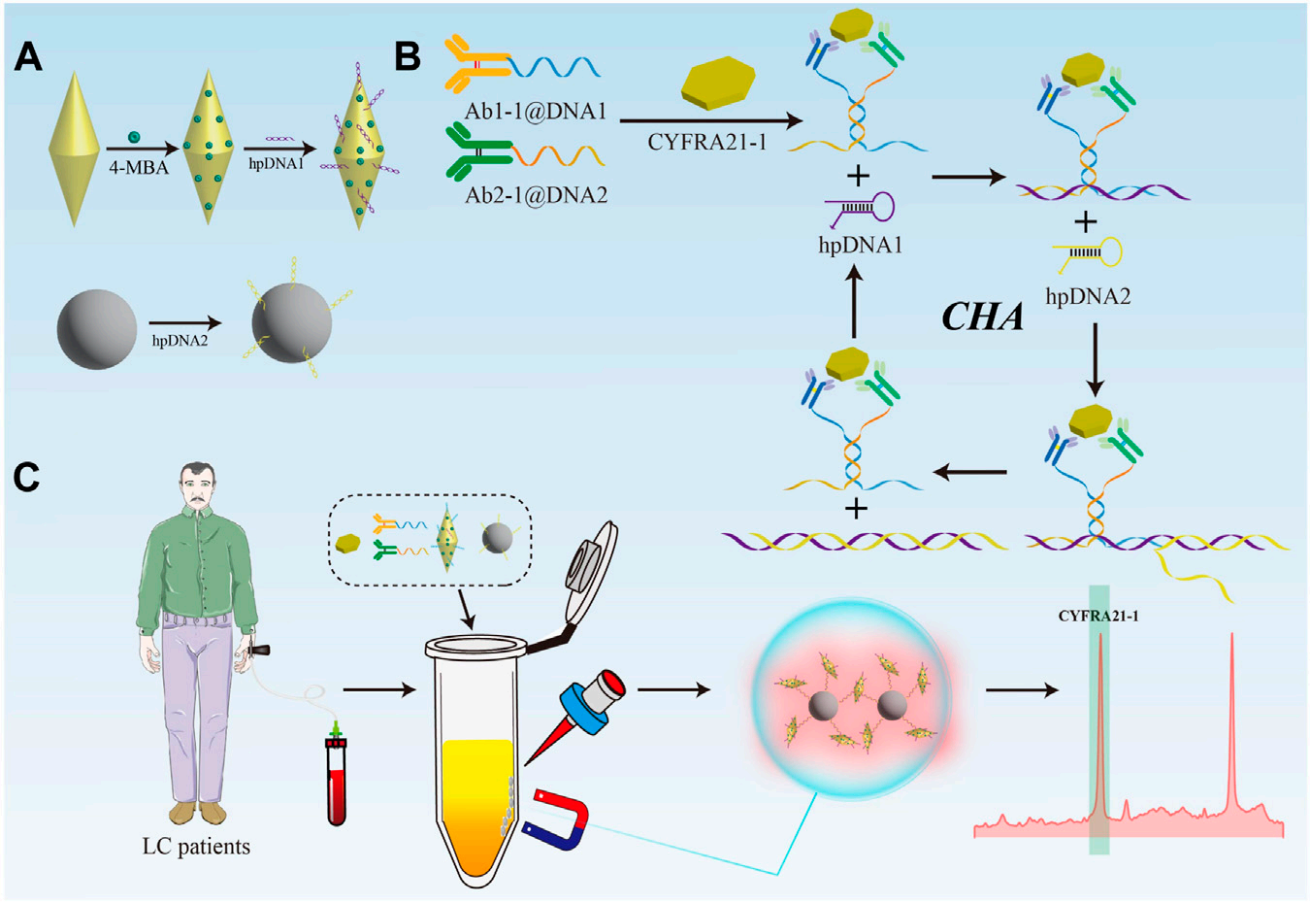
**(A)** Preparation process of SERS nanotags and capture probes. **(B)** Working mechanism of the CHA amplification. **(C)** Schematic illustration of the ultrasensitive detection of the biosensor for CYFRA21-1.

In this study, antibody-DNA conjugate was prepared to mediate the catalytic hairpin self-assembly (CHA) with magnetic sphere enrichment to achieve rapid and highly sensitive detection of CYFRA21-1 in serum for early diagnosis of LC. As shown in Figure 1A, GNBPs were modified with the Raman signal molecule 4-mercaptobenzoic acid (4-MBA) and hairpin DNA sequence 1 (hpDNA1) as SERS probes. Next, hairpin DNA sequence 2 (hpDNA2) was modified onto the surface of magnetic beads (MBs) via amide bonding as the capture probes. CHA reaction mediated by antibody-DNA conjugate was shown in Figure 1B. When CYFRA21-1 presents, it can be captured by antibody-DNA conjugate, and the significant reduction in the spatial distance allowed the DNA at the end to self-hybridize and act as a trigger for the cyclic amplification reaction. During the detection process, as shown in Figure 1C, serum samples, antibody-DNA conjugate, SERS probes and capture probes were mixed in the tube. Owing to the CHA reaction triggered by CYFRA21-1, GNBPs were continuously attached to the surface of MBs to form the SERS probes@capture probes composite structures, significantly reducing the nanogaps and accumulates the formation of abundant “hot spots”. Owing to the magnet-induced MBs aggregation, further reduction of the spacing were performed, realizing the dual-amplification of SERS signal. Depending on the degree of enhancement of the 4-MBA signal intensity, the quantitative detection of CYFRA21-1 in the serum of LC patients can be achieved.

## 2 Experimental methods

### 2.1 Chemicals

Chloroauric acid tetrahydrate (HAuCl_4_·4H_2_O), ascorbic acid (AA), bovine albumin (BSA), ethanol (CH_3_CH_2_OH), N-hydroxysuccinimide (NHS), 1-ethyl-3-(3-dimethylaminopropyl) carbodiimide (EDC), 4-mercaptobenzoic acid (4-MBA), phosphate-buffered saline (PBS), trisodium citrate dihydrate (C_6_H_5_Na_3_O_7_·2H_2_O), silver nitrate (AgNO_3_) and hexadecyl trimethyl ammonium bromide (CTAB) were purchased from Sigma-Aldrich. All reagents were provided at analytical grade and were used without further purification. CYFRA21-1, rabbit monoclonal anti-CYFRA21-1, CEA、AFP, SCC-Ag were purchased Sangon Biotech Co., Ltd. Oligonucleotides (Table 1) used were obtained from Shanghai Gene Pharma Co., Ltd.

**TABLE 1:** Sequence of oligonucleotides used in the experiment.

Name	Sequence (5′-3′)
DNA1	HS-TACGTCCAGAACTTTACCAATTTTTTTTTTTTTTTTGTGAGGGTCCATTA ACCAATG
DNA2	AGGCATTCAATACATCCTCACTTTTTTTTTTTTTTTTGGCTCTAGCGTATGCTATTG-SH
hpDNA1	HS-ACAACTGAAGTCCATTAACCAATGCCATCCTCGCAAAGGCATTCAATACATTGGTTA ATG​GAC​ATG​TAT​TGA​ATG​CCA
hpDNA2	TGC​TCT​CCA​CTT​CCA​TCC​TCG​CAA​AGG​CAT​TCA​ATA​CAT​GTC​CAT​TAA​CCA​ATG​TAT​TGA​ATG​CCT​TTG​CGA GGATGGCATTGGTTAATG-NH_2_

### 2.2 Sample collection

The serum samples used in the experiment were obtained from the Clinical Hospital of Yangzhou University. Thirty blood samples were collected from 30 LC patients and 30 healthy subjects according to routine clinical laboratory procedures (Table 2). To demonstrate the excellent performance of the proposed sensor, only the LC patients at the Ⅰ stage were selected for the measurement. The blood samples were centrifuged (15 min, 3,000 rpm) and the serum was stored frozen and set aside. All experiments were performed according to the relevant laws and obtained approval from the Ethics Committee of the affiliated hospital of Yangzhou University. Consent documents were also obtained from all human subjects.

**TABLE 2:** Basic characteristics of the participants in this study.

Groups	Healthy subjects	LC patients
Average age	27	33
Gender		
Male	14	15
Female	16	15
Sample number	30	30

### 2.3 Preparation of GNBPs

The Au seed solution was prepared by a one-step reduction method with sodium citrate (Wei et al., 2020). 80 mL of CTAB (100 mmol/L) solution and 0.8 mL of AgNO_3_ (10 mmol/L) solution were added sequentially to the beaker containing 4 mL of HAuCl_4_ (10 mmol/L) solution to obtain the growth solution. Add 1.6 mL of hydrochloric acid (1 mol/L) solution and 0.2 mL of AA (100 mmol/L) solution to the growth solution under continuous stirring. After 5 min, add 560 μL of the prepared Au seed solution to the mixed solution and placed in a water bath (28°C) for 24 h. After the color of the solution changes to dark purple, purification with ultrapure water was performed and dispersed in 20 mL of ultrapure water.

### 2.4 Preparation of SERS probes

4 mL of GNBPs solution was mixed with 200 μL of 4-MBA solution (2 mmol/L) under continuous stirring for 1 h followed by centrifugation (15 min, 7,000 rpm). Subsequently, 60 μL of hpDNA1 solution (0.1 mmol/L) was added to 80 μL of freshly prepared TECP (1 mmol/L) buffer. The activated hpDNA1 solution was mixed with 2 mL of 4-MBA modified GNBPs solution for 12 h, then mixed with 40 μL of BSA (1 wt%) solution and incubated for 120 min. Then, the solution was dispersed in PBS buffer after three times of purifications (15 min, 5,000 rpm); thus, the SERS probes were prepared. Finally, GNBP probes were washed and incubated in 10 mL of 1 wt% BSA solution for 60 min.

### 2.5 Preparation of capture probes

At room temperature, 500 μL of MBs (0.5 mg/mL) solution was mixed with 5 μL of EDC (0.1 mol/L) solution and 5 μL of NHS (0.1 mol/L) solution and incubated with shaking for 30 min (500 rpm) to activate the carboxyl groups on the surface of MBs. After adding 60 μL of activated hpDNA2 solution (0.1 mmol/L) for 12 h, 10 μL of BSA (1 wt%) was added and incubated for 120 min. Then, the MBs were separated with a magnet, the supernatant was aspirated and washed repeatedly with PBS solution.

### 2.6 SERS measurement

Serum sample was mixed with antibody-DNA conjugate, and incubated in the tube to ensure sufficient hybridization of the antibody tail DNA, followed by the addition of SERS probe solution, capture probe solution. Subsequently, a magnet was used to induce complex aggregation outside the tube wall, and the solid complex was removed and placed under Renishaw Raman microscope for testing. Test parameters: laser excitation wavelength of 785 nm, scanning range of 600–1800 cm^−1^, objective magnification of ×50, laser power of 60 mW, exposure time of 10 s. To ensure the representativeness and validity of the SERS results, the SERS spectra of 15 different spot regions were selected randomly to gained the average result. UV-Vis absorption spectra were recorded with a Mapada UV-3000PC. Transmission electron microscopy (TEM) images were taken by FEI Tecnai G2 F30 S-twin field-emission transmission electron microscopy. Scanning electron microscope (SEM) images were taken by S-4800II field emission scanning electron microscope.

## 3 Results and discussion

### 3.1 Characterization of GNBPs

**FIGURE 2:**
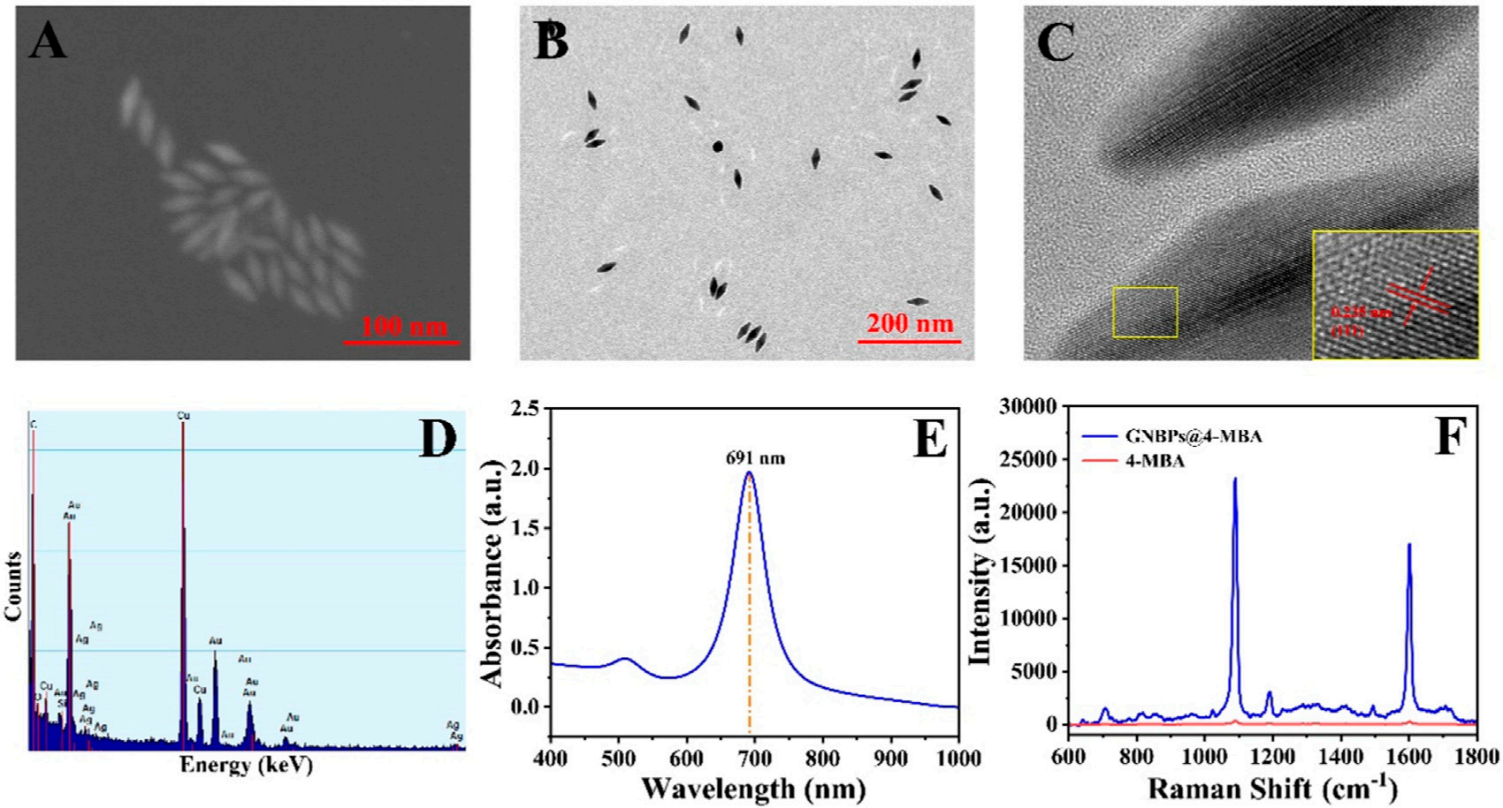
**(A)** SEM image, **(B)** TEM image, **(C)** high-resolution TEM image, **(D)** energy dispersive X-ray spectroscopy spectrum, **(E)** UV-Vis-NIR absorption spectrum of GNBPs. **(F)** Raman spectra of 4-MBA and 4-MBA-labeled GNBPs.

The morphology and structure of GNBPs were characterized by SEM and TEM. As shown in Figures 2A, B, GNBPs have two sharp tips with uniform size and good dispersion. The long diameter of GNBP is 41 nm and the short diameter is 16 nm. HRTEM image of the GNBPs (Figure 2C) shows that the lattice spacing is 0.235 nm, which corresponds to the (111) lattice plane located in the face-centered cubic crystal structure of Au. EDS spectrum of the GNBPs shown in Figure 2D reveals that the main elemental composition of GNBPs is Au and a small amount of Ag can be attributed to the underpotential deposition of Ag. Figure 2E shows a strong UV-Vis-NIR absorption peak at 693 nm due to the LSPR effect of the sharp tip on the GNBPs, while the narrow half-peak width further indicates the uniform size of the GNBPs. Figure 2F shows the SERS spectra of the Raman signal molecules 4-MBA (1 × 10^−2^ mol/L) and 4-MBA (1 × 10^−6^ mol/L)-labeled GNBPs. The characteristic peak at 1,080 cm^−1^ is generated by the in-plane cyclic respiration with υ (C-S) of 4-MBA molecular (Dziecielewski et al., 2020). The SERS signal of 4-MBA-labeled GNBPs was significantly enhanced compared to the weak signal of 4-MBA. The enhancement factor of GNBPs can be calculated according to the equation AEF=(I_SERS_/C_SERS_)/(I_RS_/C_RS_) (Yu and Cheng, 2022). Where, I_SERS_ is the SERS signal intensity of GNBPs at C_SERS_ concentration and I_RS_ is the Raman signal intensity under non-SERS conditions at C_RS_ concentration. After calculation, it is known that the enhancement factor of GNBPs is 6.67×10^5^. Therefore, GNBPs have a good SERS enhancement effect.

### 3.2 Characterization of composite structure

**FIGURE 3:**
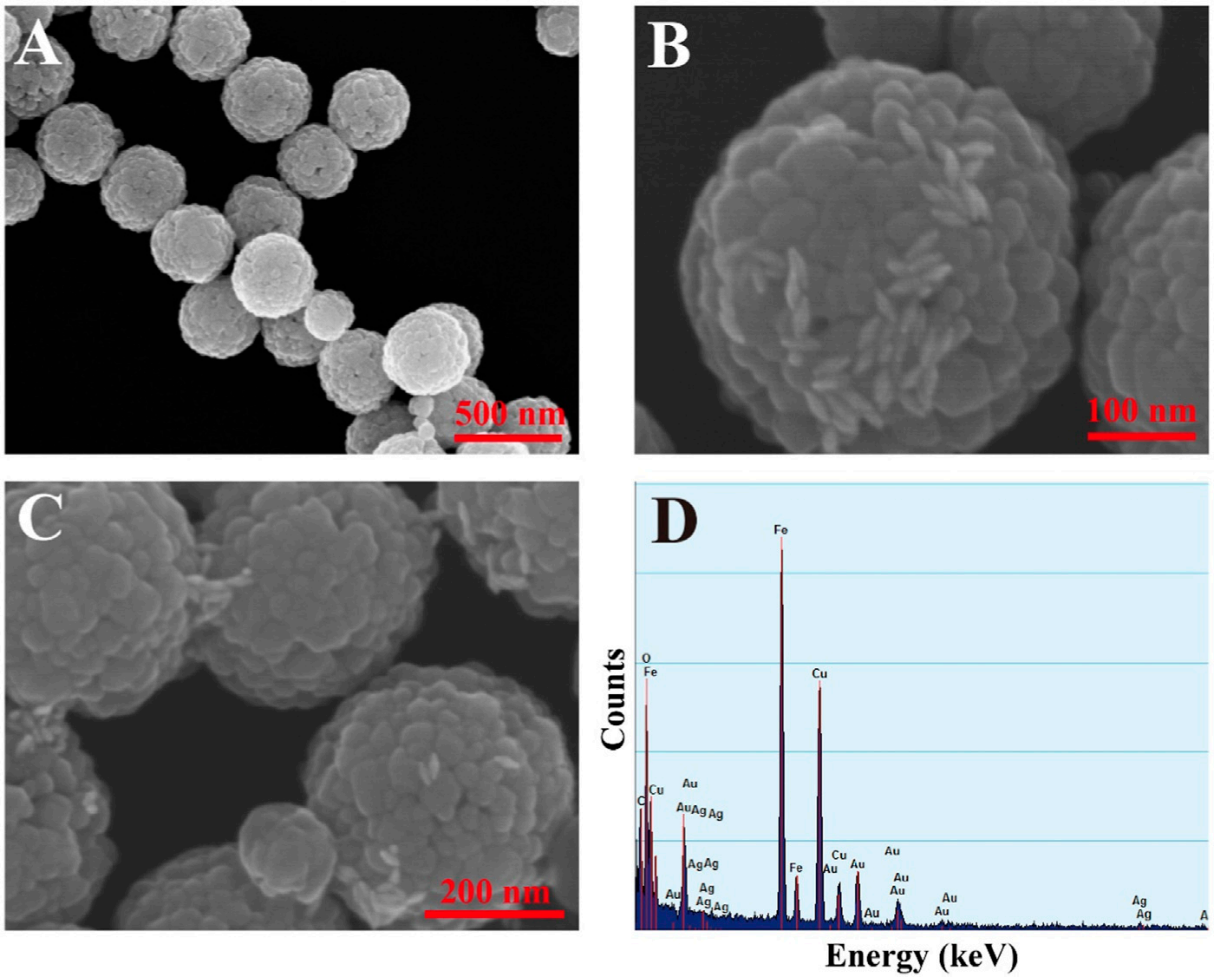
**(A)** SEM image of MBs; **(B)** SEM image of composite structures; **(C)** SEM image of composite structures; **(D)** EDS spectrum of composite structures.

The SEM image of MBs is shown in Figure 3A, which shows that the surface of MBs is rough and homogeneous in size with an average diameter of 323.9 nm. GNBPs were assembled on the surface of MBs and more aggregation of GNBPs was observed between adjacent MBs particles (as shown in Figures 3B–C), indicating that the MBs enrichment strategy and the CHA reaction could lead to significant aggregation of GNBPs and dual-amplification of the SERS signal. The EDS spectrum in Figure 3D further verifies that GNBPs are successfully aggregated on the surface of MBs. To investigate the uniformity, SERS mapping was performed and scheme of color ranging from red (maximum intensity) to blue (minimum intensity) was employed. As shown in Supplementary Figure S1, although some areas still existed as blue or red, most of them were uniformly green and the RSD was calculated to 8.29%, proving the great uniformity of the proposed SERS sensor. For an intuitive illustration, finite-difference-time-domain (FDTD) simulation was performed to confirm the spatial electric field distribution which irradiated by a beam of linearly polarized light. The complex refractive index of gold was adopted from the refractive index database in the simulation software package Au (Gold)-CRC. All geometric parameters for simulations were consistent with the average actual size. As shown in Supplementary Figure S2, high density hotspots were generated around the tips of GNBP and MBs enrichment strategy can reduce the gaps between the GNBPs, forming more hotspots to enhance the electric field intensity. This is clear evidence of the amplification of the field through interparticle plasmon coupling, resulting from the further aggregation of GNBPs caused by magnet-induced aggregation effect. Thus, the composite structure can generate numerous hotspots for the SERS signal enhancement.

### 3.3 Optimization of experimental parameters

**FIGURE 4:**
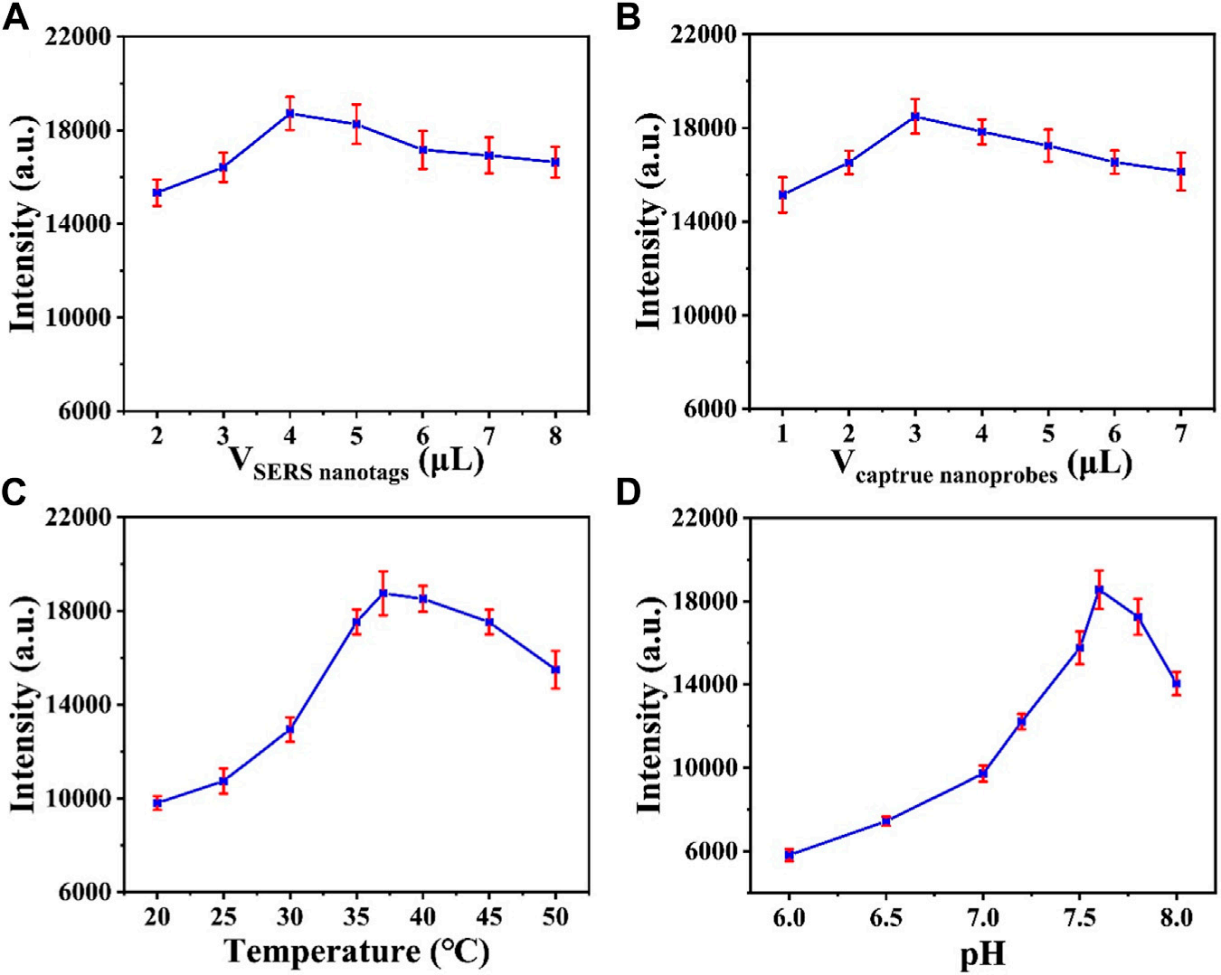
Optimization of the **(A)** volume of SERS nanotags and the **(B)** volume of capture nanoprobes. Optimization of the **(C)** reaction temperature and the **(D)** pH value.

The CHA reaction efficiency determines the amount of GNBPs captured by MBs. Figure 4A optimizes the amount of SERS probe. When the SERS probe volume is less than 4 μL, the characteristic peak intensity of 1,080 cm^1^ is significantly enhanced with increasing volume, which is due to increase of the CHA reaction efficiency caused by the increase of molecular collision chance in solution. When the SERS probe volume was greater than 4 μL, the intensity showed a decreasing trend with the increase of volume due to the fact that as the SERS probe contributes more to the background signal than to the SERS signal. Therefore, the optimal SERS probe amount is 4 μL. Similarly, the amount of capture probe can be optimized to 3 μL (Figure 4B). The reaction temperature is critical for hairpin DNA hybridization in CHA reactions. As shown in Figure 4C, the intensity of the 1,080 cm^1^ characteristic peak increases with increasing temperature and reaches a maximum at 37°C. Figure 4D optimizes the pH of the reaction and it can be observed that the reaction efficiency is best when the pH is 7.5; therefore, the optimum pH for the reaction is 7.5.

### 3.4 Sensing performance analysis

**FIGURE 5:**
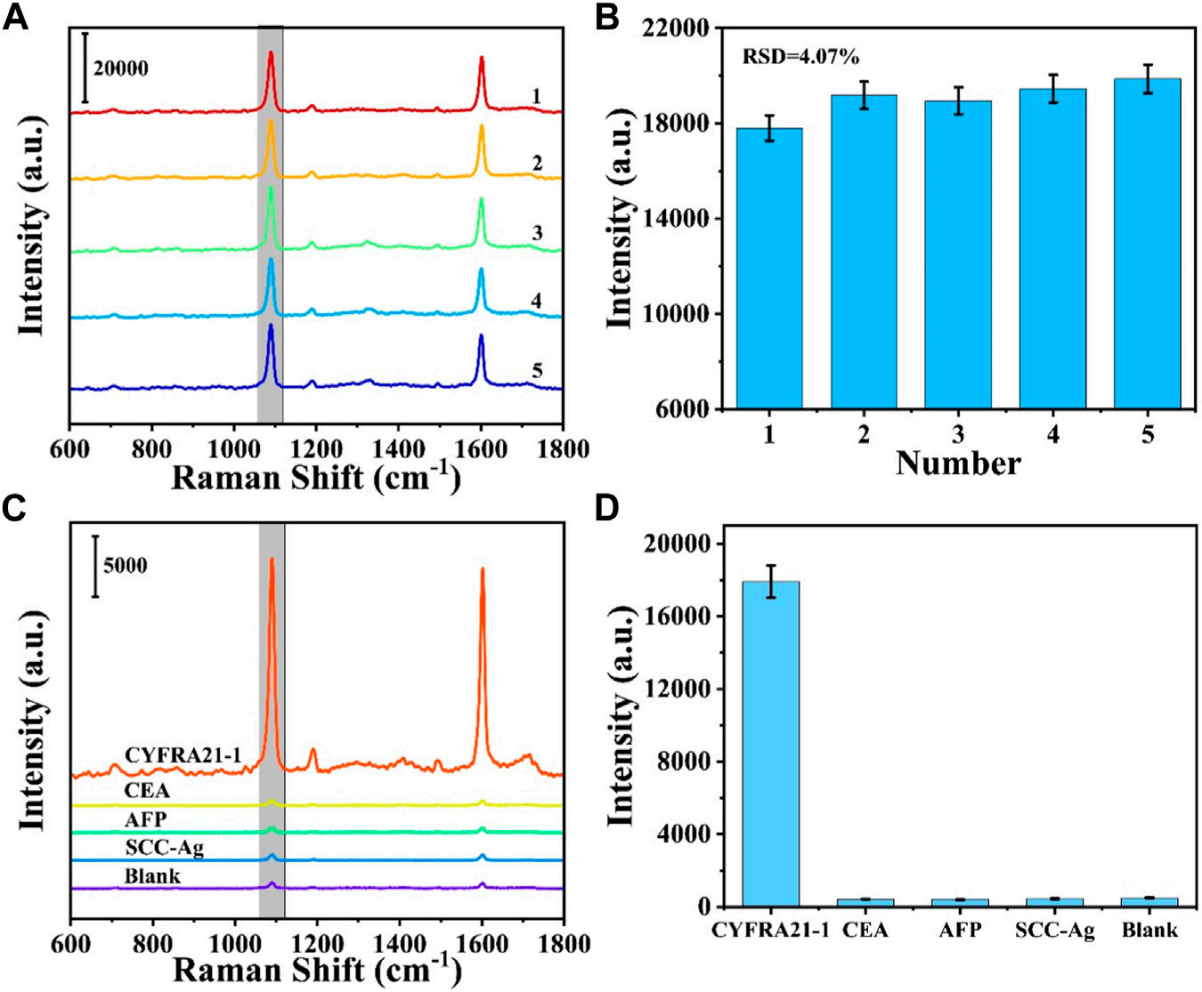
**(A)** SERS spectra from parallel measurements on five independent SERS sensors and **(B)** corresponding scatter diagram of peak intensities at 1,080 cm^−1^. **(C)** SERS spectra of different targets: CYFRA21-1, CEA, AFP, SCC-Ag. **(D)** Corresponding histogram of peak intensities at 1,080 cm^−1^.

Five different batches of prepared SERS sensors were selected for the detection of CYFRA21-1, and the detection reproducibility of the SERS sensors was verified. As shown in Figure 5A, the five SERS spectra did not differ significantly in shape. Figure 5B shows the intensity of the five SERS spectra at 1,080 cm^1^ characteristic peak with a relative standard deviation (RSD) of 4.07%, indicating that the prepared SERS sensor has excellent reproducibility. To investigate the specificity of the SERS sensor, CEA, AFP and SCC-Ag were selected as interferents. CYFRA21-1 solution (1 μg/mL) and CEA, AFP, SCC-Ag solution (10 μg/mL) and blank control were detected using the SERS sensor, respectively. As shown in Figures 5C, D, CYFRA21-1 exhibited significant characteristic peaks compared with the interferer and blank control, indicating the good specificity of the prepared SERS sensor.

### 3.5 Sensing performance analysis

**FIGURE 6:**
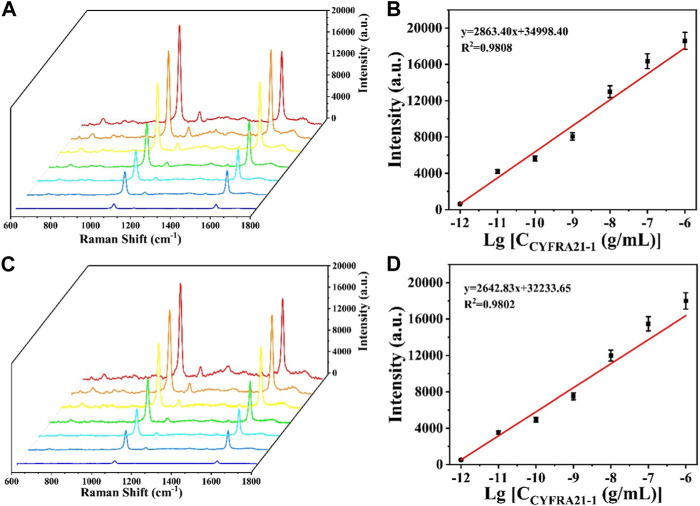
**(A)** SERS spectra of CYFRA21-1 with different concentrations in PBS buffer; **(B)** calibration curve between peak intensity at 1,080 cm^−1^ and logarithm of CYFRA21-1 concentration; **(C)** SERS spectra of CYFRA21-1 with different concentrations in serum; **(D)** calibration curve between peak intensity at 1,080 cm^−1^ and logarithm of CYFRA21-1 concentration.

CYFRA21-1 was dispersed into PBS buffer and diluted to different concentrations (1 pg/mL, 10 pg/mL, 100 pg/mL, 1 ng/mL, 10 ng/mL, 100 ng/mL, and 1 μg/mL). Detection of different concentrations of CYFRA21-1 was performed with the prepared SERS sensor and the working curve of CYFRA21-1 versus the amount of change in SERS signal intensity was plotted based on the signal of the 4-MBA at the characteristic peak at 1,080 cm^1^. As shown in Figure 6A, the SERS signal intensity gradually increased with the increase of CYFRA21-1 concentration. The concentration-intensity correction curve in logarithmic coordinates was obtained by taking the logarithm of CYFRA21-1 concentration as the horizontal coordinate and the SERS signal intensity at the 1,080 cm^1^ characteristic peak as the vertical coordinate (Figure 6B). It can be observed that the SERS signal intensity at the characteristic peak of 1,080 cm^1^ showed a good linear relationship with the logarithm of CYFRA21-1 concentration in the range of 1 pg/mL∼1 μg/mL. The linear regression equation was y = 2,863.40x+34,998.40 (*R* ^2^ = 0.9808) and the limit of detection for CYFRA21-1 in PBS was calculated to 0.69 pg/mL. Similarly, to investigate the detection performance of CYFRA21-1 in serum with the SERS sensor, CYFRA21-1 was dispersed into serum and diluted to different concentrations (1 pg/mL, 10 pg/mL, 100 pg/mL, 1 ng/mL, 10 ng/mL, 100 ng/mL, and 1 μg/mL). As shown in Figures 6C, D, the SERS signal intensity showed a good linear relationship with the logarithm of CYFRA21-1 serum concentration. The linear regression equation was y = 2,642.83x+32,233.65 (*R* ^2^ = 0.9802). The detection limit of the SERS sensor for CYFRA21-1 in serum was calculated to be 0.76 pg/mL. As shown in Table 3, the sensitivity of the SERS sensor based on magnetic sphere enrichment and CHA signal amplification was superior to other protein marker detection methods. These results indicate that the SERS sensor can be used for the quantitative analysis of CYFRA21-1 with excellent sensitivity.

**TABLE 3:** Comparison of the proposed SERS sensor with other methods in protein biomarker detection.

Strategy	Target	LOD	Ref
Fluorescence	BSA	3 ng/mL	Li et al. (2013)
SERS (label-free)	HG	0.4 ng/mL	Arabi et al. (2021)
SERS	PSA	10 pg/mL	Gao et al. (2019)
SERS-Aptamer	CRP	0.72 pg/mL	Kim et al. (2022)
SERS-CHA	CYFRA21-1	0.83 pg/mL	This work

### 3.6 Clinical sample analysis

**FIGURE 7:**
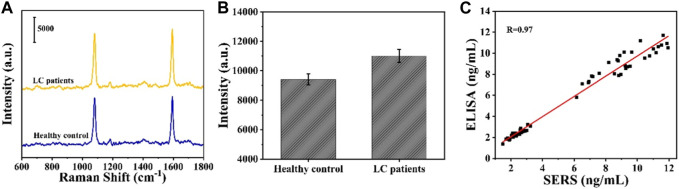
**(A)** Average SERS spectra in serum obtained from LC patients and healthy controls; **(B)** corresponding scatter diagram of peak intensities at 1,080 cm^−1^; **(C)** Linear fitting in the measured expression levels of CYFRA21-1 between SERS and ELISA.

In order to investigate the accuracy of the SERS sensor in clinical application, CYFRA21-1 expression levels in the serum of healthy individuals and LC patients were measured via the SERS sensor. Figures 7A, B show the average SERS spectra of serum SERS and the signal intensity at the characteristic peak of 1,080 cm^1^ in 30 healthy individuals and 30 LC patients, respectively, and it can be seen that the intensity at the characteristic peak of 1,080 cm^1^ in LC patients is significantly higher than that in normal individuals. The expression level of CYFRA21-1 was calculated by substituting the intensity at the 1,080 cm^1^ peak in the SERS spectra of 30 healthy subjects and 30 LC patients into the linear regression equation (Supplementary Table S1). To verify the accuracy of the SERS assay results, the SERS assay results were linearly fitted to the ELISA results, and the Pearson correlation coefficient was calculated to be 0.97, indicating that the results of the two assays were highly correlated (Figures 7C). Therefore, the SERS sensor has good accuracy and broad application prospect for clinical diagnosis of LC.

## 4 Conclusion

A novel SERS sensor was prepared for the detection of CYFRA21-1 in serum of LC patients. The CHA reaction based on antibody-DNA conjugate can capture GNBPs onto the surface of MBs, and the MBs enrichment strategy can significantly reduce the particle gap and form a large number of “hot spots,” realizing the dual-amplification of the SERS signal. The SERS sensor has the advantages of high sensitivity, excellent reproducibility and good specificity, and the detection limits of CYFRA21-1 in PBS buffer and serum were 0.69 pg/mL and 0.76 pg/mL, respectively. CYFRA21-1 expression levels in the serum of LC patients were significantly higher than those of healthy subjects. In addition, the ELISA results verified the accuracy of the SERS assay. In conclusion, this study provides a new idea for the construction of SERS sensor for protein biomarker detection and proposes a simple and reliable new method for the clinical diagnosis of early LC.

## Data Availability

The raw data supporting the conclusion of this article will be made available by the authors, without undue reservation.
