# Supplementary material for: Highly sensitive detection of CYFRA21-1 with a SERS sensing platform based on the MBs enrichment strategy and antibody-DNA-mediated CHA amplification
Source: Front Bioeng Biotechnol. 2023 Aug 10;11:1251595. doi: 10.3389/fbioe.2023.1251595 (PMC10449459; doi:10.3389/fbioe.2023.1251595)
Supplement: Supplementary file 1 [file DataSheet1.docx]

**Highly sensitive detection of CYFRA21-1 with a SERS sensing platform based on the MBs enrichment strategy and antibody-DNA-mediated CHA amplification**

Xiaotao Bao^1^, Shiyi wang^1^, Xiaoyan Liu^2^, Guang Li^1^*

^1^Department of Otorhinolaryngology Head and Neck Surgery, The Affiliated Hospital of Yangzhou University, Yangzhou University, Yangzhou, 225001, P. R. China.

^2^Department of Otorhinolaryngology Head and Neck Surgery, Xishan People’s Hospital of Wuxi City, Wuxi, 214000, P. R. China.

*Correspondence: entliguang@163.com


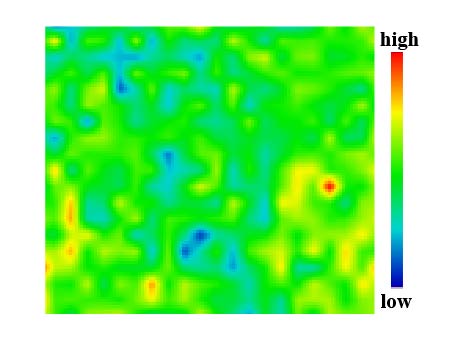


**Fig. S1** SERS mapping of the composite structures.


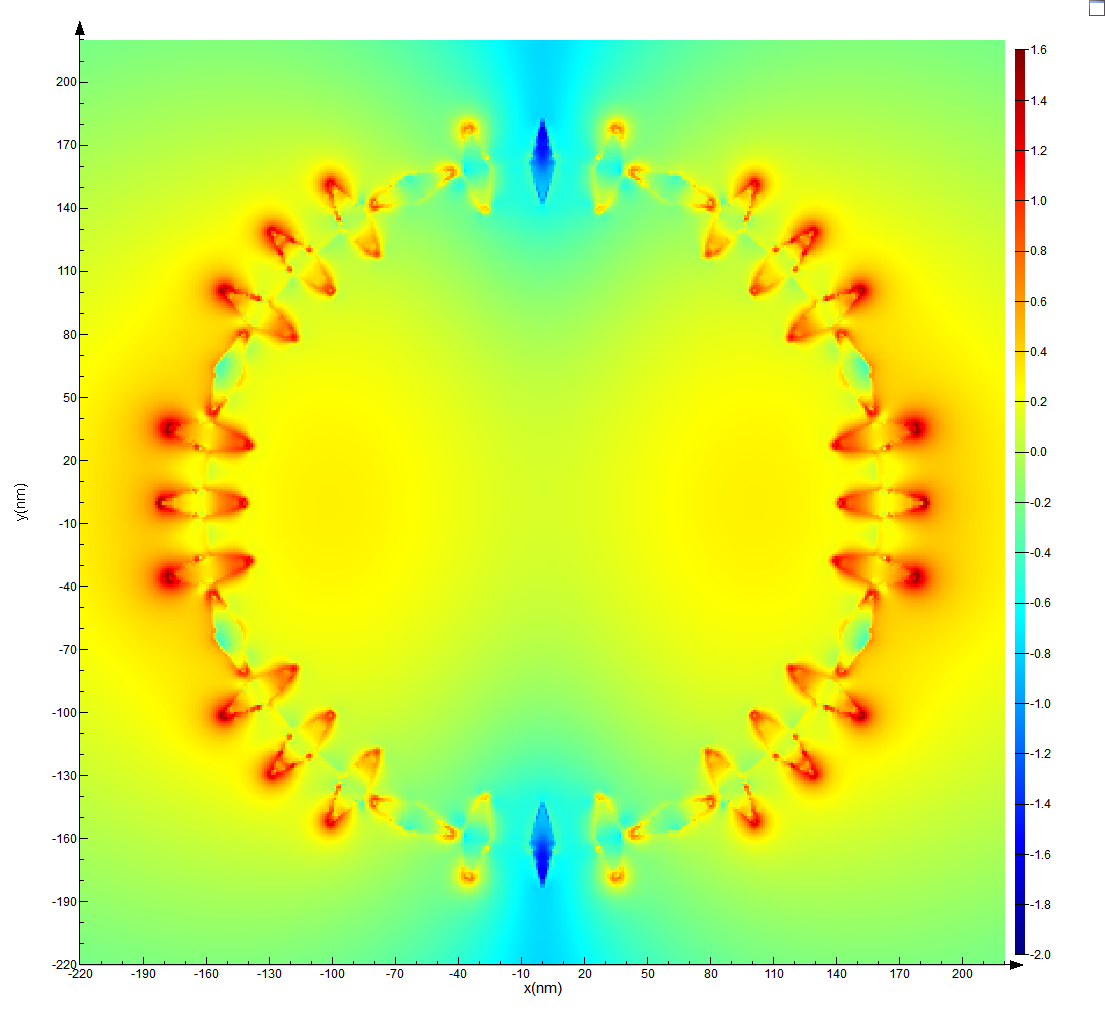


**Fig. S2** Simulation results of the distribution of the relative electric field of the composite structure for 785 nm excitation.

**Table S1** Comparison of SERS and ELSIA method in clinical samples

| Samples | Healthy subjects (ng/mL) | | | LC patients (ng/mL) | | |
| --- | --- | --- | --- | --- | --- | --- |
|  | SERS | ELISA | Relative error (%) | SERS | ELISA | Relative error (%) |
|  | 2.62 | 2.45 | 6.49 | 11.64 | 10.78 | 7.39 |
|  | 1.94 | 1.84 | 5.15 | 11.02 | 10.42 | 5.44 |
|  | 3.01 | 2.88 | 4.32 | 11.88 | 10.91 | 8.16 |
|  | 2.04 | 2.22 | -8.82 | 8.82 | 8.18 | 7.26 |
|  | 2.61 | 2.87 | -9.96 | 6.91 | 7.22 | -4.49 |
|  | 1.79 | 1.93 | -7.82 | 10.19 | 11.18 | -9.72 |
|  | 2.32 | 2.12 | 8.62 | 8.19 | 8.81 | -7.57 |
|  | 2.57 | 2.71 | -5.45 | 9.28 | 8.57 | 7.65 |
|  | 2.89 | 2.69 | 6.92 | 6.51 | 7.09 | -8.91 |
|  | 1.68 | 1.75 | -4.17 | 7.19 | 7.78 | -8.21 |
|  | 2.29 | 2.42 | -5.68 | 11.32 | 10.63 | 6.10 |
|  | 2.76 | 2.54 | 7.97 | 6.16 | 5.81 | 5.68 |
|  | 2.81 | 2.65 | 5.69 | 8.71 | 9.37 | -7.58 |
|  | 2.33 | 2.13 | 8.58 | 9.37 | 8.73 | 6.83 |
|  | 2.13 | 2.02 | 5.16 | 9.64 | 10.12 | -4.98 |
|  | 2.55 | 2.39 | 6.27 | 7.59 | 8.09 | -6.59 |
|  | 2.75 | 2.57 | 6.55 | 8.56 | 8.06 | 5.84 |
|  | 2.43 | 2.28 | 6.17 | 9.16 | 9.91 | -8.19 |
|  | 2.11 | 2.28 | -8.06 | 11.95 | 11.23 | 6.03 |
|  | 2.67 | 2.48 | 7.12 | 6.93 | 7.32 | -5.63 |
|  | 2.35 | 2.17 | 7.66 | 7.11 | 7.82 | -9.99 |
|  | 3.24 | 3.07 | 5.25 | 8.78 | 9.28 | -5.69 |
|  | 2.22 | 2.35 | -5.86 | 10.48 | 9.55 | 8.87 |
|  | 1.91 | 1.76 | 7.85 | 10.78 | 9.76 | 9.46 |
|  | 3.06 | 3.22 | -5.23 | 8.95 | 8.27 | 7.60 |
|  | 2.14 | 2.28 | -6.54 | 9.55 | 8.78 | 8.06 |
|  | 2.11 | 2.29 | -8.53 | 8.89 | 9.77 | -9.90 |
|  | 1.48 | 1.36 | 8.11 | 11.52 | 10.78 | 6.42 |
|  | 1.73 | 1.86 | -7.51 | 9.22 | 8.83 | 4.23 |
|  | 2.54 | 2.36 | 7.09 | 11.19 | 10.66 | 4.74 |
